# Une complication rare et grave du traitement par l’acénocoumarol: l’hémorragie intra-alvéolaire

**DOI:** 10.11604/pamj.2019.33.160.18708

**Published:** 2019-07-03

**Authors:** Anas Loutfi, Sara Chibane, Abdenasser Drighil, Leila Azzouzi, Rachida Habbal

**Affiliations:** 1 Service de Cardiologie, CHU Ibn Rochd, Casablanca, Maroc

**Keywords:** Hémorragie intra-alvéolaire, anti-vitamines K, diagnostic, prise en charge, pronostic, Intra-alveolar bleeding, anti-vitamins K antagonists, diagnosis, management, prognosis

## Abstract

L'hémorragie intra-alvéolaire est une urgence médicale rare et grave ayant de nombreuses étiologies. Nous rapportons ainsi le cas clinique d'un patient qui pourrait contribuer à élargir la littérature sur ce sujet. Le patient est un homme âgé de 62 ans ayant un antécédent de fibrillation auriculaire sous anti-vitamines K et admis dans un tableau de dyspnée d'apparition brutale avec hémoptysie et notion d'automédication par anti-inflammatoires non stéroïdiens. La radiographie et le scanner thoracique ont montré des opacités alvéolaires bilatérales diffuses. Au bilan d'hémostase de l'admission, l'INR était incoagulable. Le diagnostic d'hémorragie intra-alvéolaire était suspecté cliniquement et radiologiquement puis confirmé par l'endoscopie bronchique avec lavage broncho-alvéolaire (LBA) retrouvant un liquide uniformément hémorragique. Les études précédentes présentant des cas similaires secondaires à la prise d'anti-vitamines K sont rares. En conclusion, Il parait très important de souligner l'intérêt d'une surveillance stricte et optimale clinico-biologique des patients traités en anti-vitamines K afin d'éviter un surdosage pouvant contribuer à un accident hémorragique grave engageant le pronostic vital.

## Abstract (en)

Intra-alveolar bleeding is a rare and severe medical emergency due to numerous causes. We report the clinical case of a patient who could contribute to extend the literature on this subject. The study included a 62-year old man, with a history of a trial fibrillation, under anti-vitamins K antagonist admitted with dyspnoea of sudden onset associated with haemoptysis and practising self-medication using non-steroidal anti-inflammatory drugs. X-rays and chest scan showed diffuse bilateral alveolar opacities. Haemostatic screening tests on admission showed non-coagulable INR. The diagnosis of intra-alveolar bleeding was clinically and radiologically suspected and then confirmed by bronchial endoscopy with broncho-alveolar lavage (BAL) which detected uniformly hemorrhagic liquid. Previous studies of similar complications occurring after anti-vitamins K antagonists assumption are rare. In conclusion, it seems very important to emphasize the interest of strict and optimal clinico-biological monitoring of patients treated in anti-vitamins K antagonists to avoid an overdose which could contribute to a life-threatening severe haemorrhagic event.

## Introduction

L'hémorragie intra-alvéolaire (HIA) est une urgence thérapeutique rare pouvant engager le pronostic vital. Elle se réfère à un syndrome clinique caractérisé par un saignement important provenant de la microcirculation de l'acinus pulmonaire [1]. Les lésions peuvent toucher toutes les structures de la cloison alvéolo-capillaire: épithélium, membrane basale et/ou endothélium. Classiquement, il se manifeste par la triade clinique de dyspnée, hémoptysie et anémie, avec opacités infiltrantes diffuses radiologiques. Les étiologies de l'HIA sont nombreuses et variées d'origines immunes ou non immunes incluant: infections pulmonaires, embolie pulmonaire, maladie de Wegener, syndrome de *Goodpasture*, maladie de Churg et Strauss, lupus érythémateux disséminé et maladie de Behçet [2, 3]. L'hémorragie intra-alvéolaire (HIA) due aux anti-vitamines K a été rarement rapportée dans la littérature [4-6]. Dans cet article, nous rapportons le cas d'un patient ayant une fibrillation auriculaire permanente traitée par anti-vitamines K admis dans notre service avec hémoptysie et dyspnée. L'hémorragie intra-alvéolaire (HIA) a été suspectée cliniquement et les résultats radiologiques ont été confirmés par bronchoscopie avec lavage broncho-alvéolaire.

## Patient et observation

**Figure 1:**
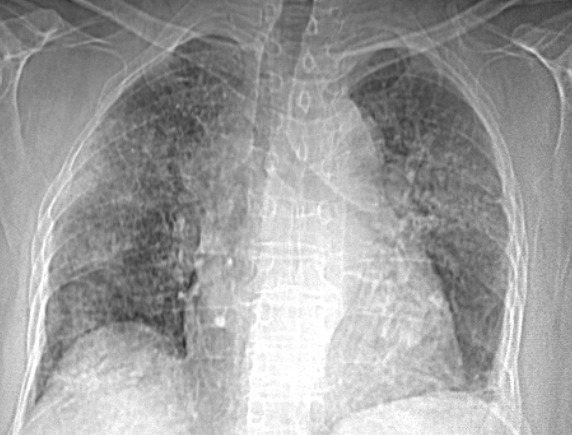
Radio thoracique de face révélant des opacités alvéolaires bilatérales diffuses

**Figure 2:**
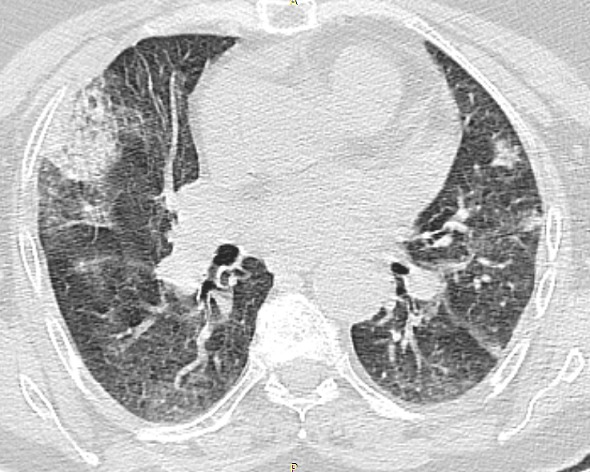
TDM thoracique en coupe parenchymateuse révélant une condensation alvéolaire avec des opacités en verre dépoli

Le patient est un homme âgé de 62 ans, hypertendu, ayant un antécédent de fibrillation auriculaire sous anti-vitamines K depuis 13 ans bien suivi. Il s'est présenté à l'hôpital dans un tableau de dyspnée d'apparition brutale stade II NYHA, hémoptysie avec notion d'automédication par Ibuprofène depuis plusieurs jours. À son admission, le patient était apyrétique, tachypnéique à 28 cycles/min, tachycarde à 126bpm, une pression artérielle à 134/79mmHg, saturation artérielle en oxygène à 94% avec des râles crépitants diffus aux deux hémi champs pulmonaires. Au bilan biologique, nous avons noté une anémie à 7,8g/dl hypochrome microcytaire, des globules blancs à 10230/mm^3^, des plaquettes à 260000/mm^3^, un INR incoagulable, une créatininémie à 10,3g/l estimant le débit de filtration glomérulaire à 77ml/min/1,73m^2^ (Formule MDRD simplifiée), une albuminémie à 34g/l, protidémie à 63g/l. L'examen de la bandelette urinaire s'est révélé négatif sans hématurie ni macroalbuminurie. Les taux sériques des p-ANCA, c-ANCA, Ac anti-MBG, AC anti-DNA ainsi que le facteur rhumatoïde se sont révélés négatifs. L'échocardiographie transthoracique a montré une fonction ventriculaire normale et aucune valvulopathie. Le cliché standard du thorax a révélé à la phase d´état des opacités alvéolaires bilatérales diffuses (Figure 1), ce qui nous a incité à compléter par une TDM thoracique retrouvant des lésions alvéolaires multiples bilatérales (Figure 2). Enfin, une endoscopie bronchique avec lavage broncho-alvéolaire (LBA) fut réalisé retrouvant un liquide uniformément hémorragique avec cultures revenues stériles.

## Discussion

Les accidents hémorragiques aux AVK représentent un véritable problème de santé publique. En France, ils sont la première cause d'hospitalisation pour accident iatrogène. Au Royaume Uni, ils viennent en troisième position [7]. L'hémorragie intra-alvéolaire (HIA) attribuable à l'utilisation des anti-vitamines K, est une complication rare potentiellement mortelle par insuffisance respiratoire aiguë [8, 9]. Elle est définie par la présence d'hématies dans les lumières alvéolaires constatée à l'endoscopie bronchique par un lavage broncho alvéolaire (LBA), ou par une biopsie pulmonaire après avoir éliminé les principales pathologies pouvant être incriminées dans la survenue de cette manifestation, dont les maladies auto-immunes (Maladie de Wegener, syndrome de *Goodpasture*, maladie de Churg et Strauss, lupus érythémateux disséminé, maladie de Behçet et syndrome des anticorps antiphospholipides), les infections pulmonaires, l'embolie pulmonaire, hypertension artérielle pulmonaire, les expositions toxiques, les réactions aux drogues (amiodarone, méthotrexate …), la sténose mitrale et l'hémosidérose pulmonaire idiopathique [10, 11]. Le traitement par acénocoumarol est guidé par le rapport international normalisé (INR). Plusieurs facteurs interviennent dans l'augmentation du risque hémorragique chez les patients sous anti-vitamines K comme l'utilisation de certains antibiotiques, antifongiques ou anti-inflammatoires, l'aspirine, l'héparine, l'amiodarone, les IPP, les anticonvulsivants, l'allopurinol,la consommation d'alcool, l'âge avancé, les insuffisances hépatiques et rénales, le diabète ainsi que l'alimentation [12, 13]. Les études précédentes présentant des cas d'hémorragies intra-alvéolaires secondaires à la prise d'anti-vitamines K sont rares. Nous rapportons ainsi le cas clinique de notre patient qui pourrait contribuer à la littérature sur ce sujet. Nous proposons ainsi une surveillance stricte et optimale clinico-biologique de ces patients afin d'éviter un surdosage pouvant contribuer à un accident hémorragique grave. L'hémorragie intra-alvéolaire est un diagnostic rare à évoquer car le risque de mortalité est important s'il n'est pas détecté rapidement et traité précocement.

## Conclusion

L'hémorragie intra-alvéolaire (HIA) liée à l'utilisation de l'acénocoumarol est un événement rare mais pouvant être létal du fait de sa présentation initiale et son évolution imprévisibles. Sa détection doit être rapide et le traitement agressif tout en recherchant les facteurs de risque ainsi que la probable étiologie pouvant être à l'origine de cette manifestation.

## Conflits d’intérêts

Les auteurs ne déclarent aucun conflit d'intérêts.
